# A century of climate warming results in growing season extension: Delayed autumn leaf phenology in north central North America

**DOI:** 10.1371/journal.pone.0282635

**Published:** 2023-03-03

**Authors:** Kellen Calinger, Peter Curtis

**Affiliations:** Evolution, Ecology, and Organismal Biology, Aronoff Laboratory, The Ohio State University, Columbus, Ohio, United States of America; Tennessee State University, UNITED STATES

## Abstract

Shifts in the timing of key leaf phenological events including budburst, foliage coloration, and leaf fall have been observed worldwide and are consistent with climate warming. Quantifying changes in growing season length (GSL) because of shifts in both spring and autumn leaf phenology is crucial for modeling annual net ecosystem carbon uptake. However, a lack of long-term autumn phenology datasets has prevented assessment of these growing season level changes. We investigated shifts in growing season length, budburst, foliage coloration, and leaf fall over the past century in seven native hardwood species using a historic leaf phenology dataset collected in Wauseon, OH from 1883–1912 paired with contemporary observations. Using long-term meteorological data, we investigated temperature and precipitation trends over 130 years. Finally, we correlated spring and fall phenophases with monthly temperature and precipitation variables from the twelve months preceding that phenophase using historical meteorological data. We found significant extension of growing season length over the past century in five of the seven study species (ANOVA, *p* < 0.05) which resulted primarily from delayed foliage coloration rather than from earlier budburst in contrast to the few other studies assessing total GSL change. Our results suggest that most of the leaf phenological studies that investigate only budburst are disregarding crucial information about the end of the growing season that is essential for accurately predicting the effects of climate change in mixed-species temperate deciduous forests.

## Introduction

Overwhelming evidence indicates that climate change has already resulted in significant shifts in the timing of crucial plant phenological events such as budburst in the spring, flowering, and autumn leaf coloration and fall [1–4]. Generally, warmer temperatures are associated with advancement of budburst and, more variably, delay of autumn leaf senescence [5–7]. Shifts in leaf phenological events are likely to be particularly important drivers of altered ecosystem function in temperate forests of the northern hemisphere with their strongly seasonal growing periods [1, 8].

Altered timing of spring and fall leaf phenology exerts significant control over ecosystem carbon sequestration by altering growing season length [9]. Increased growing season length because of warming allows a longer period of photosynthetic activity and carbon absorption [10] and has been identified as a major driver of the increasing terrestrial carbon sink [9–11]. However, while extension of the growing season through earlier budburst has been linked consistently with increased carbon storage, the effects of delayed autumn leaf senescence are more varied. For example, carbon uptake increased with growing season elongation as a result of both earlier spring and later autumn leaf phenology across 21 temperate and boreal forest study sites [12]. In direct opposition to these findings, growing season extension through autumn warming and the resultant delay in leaf senescence was shown to cause net carbon losses as ecosystem respiration rates increased more than gross primary productivity across 22 broadleaf and evergreen forest sites [13, 14]. Despite these important ecological effects, species-specific leaf phenological responses to climate change that drive alteration of growing season length and therefore ecosystem carbon storage are relatively understudied [15, 16].

Species living in the same community show markedly different timing of budburst and leaf senescence despite the identical trade-off scenarios of their shared environment [17]. These differences in leaf phenological timing indicate species-specific variation in drivers of leaf phenology and/or differing sensitivity to climatological variables like temperature and precipitation [1, 6, 18, 19]. Generally, budburst is driven by warming temperatures following a chilling period and by photoperiod [20] although the importance of each varies widely between species [21] and specific drivers of budburst for most species remain unknown [22]. For example, budburst in maple (*A*. *pseudoplatanus*), beech (*F*. *sylvatica*), ash (*F*. *excelsior*), and oak (*Q*. *petraea*) was significantly advanced by warmer temperatures in March, April, and May [1]. However, ash was 3-fold more responsive to spring warming than beech advancing budburst by 6.6 and 1.9 days/°C, respectively. The role of photoperiod in driving budburst is even more variable between species with some having no photoperiod requirement (eg. *Betula* spp, *Populus* spp [23]) while others will not burst bud irrespective of warming if photoperiod requirements have not been met (ie. *Fagus grandifolia*, [24]). Hypotheses regarding autumn phenological drivers are mixed with different studies reporting opposite effects of temperature while the role of precipitation remains unclear [1, 18, 25]. For example, one study [1] reported significant delays in leaf senescence in oak and beech (*Quercus petraea* and *Fagus sylvatica*) with warming in the months immediately preceding senescence. In contrast, another study [24] found that warmer summer and autumn temperatures resulted in advancement of leaf fall in oak, birch, and poplar (*Quercus robur*, *Betula pubescens*, and *Populus canescens*, respectively). In other species, leaf senescence shows no interannual variation suggesting that photoperiod is the primary driver of senescence in these species (*Acer pseudoplatanus* and *Fraxinus excelsior*, [1]). One of the few studies to assess precipitation as a potential driver of autumn phenology found mixed responses among species [17]. Some showed advancement or delay of leaf coloration and fall with increasing or decreasing precipitation throughout the spring, summer, and fall preceding senescence [17]. Further, individual phenophases within a species, like leaf coloration and leaf fall, show distinct responses to both temperature and precipitation drivers [15, 17]. Given the significant interspecific variation in responsiveness to climatological drivers of spring and autumn phenology, individual species likely will have distinct responses to further climate warming. Our limited knowledge of species-specific alteration of both spring and fall leaf phenology and the inconsistent relationships reported in the literature [26] restricts our ability to accurately predict the effects of further warming on total growing season length, species interactions, species persistence in their current ranges, and ecosystem C storage dynamics [1, 27].

One major impediment to assessing species-specific changes in leaf phenology across the growing season is the dearth of multi-decadal datasets of autumn leaf phenology [18, 28]. The lack of autumn phenophase data limits our ability to quantify changes in total growing season length, a crucial variable for understanding future ecosystem C dynamics [1]. The majority of studies that have assessed changes in growing season length occurred in European ecosystems where historic data sets and long-term phenology monitoring programs are more common [1, 2, 29]. Given the relative rarity of historic phenophase data, the species-specific leaf phenological responses of trees in North American temperate forest ecosystems remain relatively understudied [20].

To address our knowledge gaps regarding phenological responses to climate change, we used a historic record of spring and fall leaf phenology coupled with modern phenology observations to assess species-specific shifts in leaf phenology in a mixed-species temperate deciduous forest in north-central North America. The objectives of this study were: 1) to quantify species-specific shifts in budburst, foliage coloration, and leaf fall over the past 130 years in seven woody species, 2) to evaluate the importance of spring *versus* fall phenological shifts in altering total growing season length, 3) to quantify regional long-term temperature and precipitation changes over roughly the past 130 years in north-central North America, and 4) to determine species-specific relationships between budburst, foliage coloration, and leaf fall with climatological drivers.

## Methods

### I. Study site and study species

All observations were conducted in Wauseon, Fulton County, located in northwest Ohio (41°33’8”N, 84°8’21”W). Wauseon was selected as our study site because of a rich historical phenological and meteorological dataset collected by Thomas Mikesell, a farmer and Wauseon resident, from 1883–1912. The Wauseon landscape has been agriculturally dominated since at least the 1880’s and is interspersed with hedgerows and woodlots [30]. Topographical variation is considered too minimal to cause substantial microclimate variability [31].

The seven species chosen for observation were *Ulmus americana* (American elm), *Juglans nigra* (black walnut), *Quercus alba* (white oak), *Quercus velutina* (black oak), *Populus deltoides* (eastern cottonwood), *Rhus typhina* (staghorn sumac), and *Sassafras albidum* (sassafras). All species have broad distributions across the eastern temperate forest and none are near the northern or southern edges of their ranges in Wauseon. No IRB, IACUC, or ethics committee approval was required for this study as it was entirely observational and constituted no risk for any living organism. No organisms were harmed or altered by our observations which were exclusively visual.

### II. Long-term meteorological data

We used mean monthly temperature (°C) and total monthly precipitation (cm) data from the U.S. Historical Climatology Network’s (USHCN) Wauseon station (temperature and precipitation data available from 1892–2022 and 1883–2021, respectively [32]) to calculate long-term temperature and precipitation trends. Simple linear regressions of mean monthly temperatures (from 1892–2014) and total monthly precipitation values (from 1883–2013) versus time (years) were used to calculate the rate of change in temperature and precipitation (slope of the regression) and total change over the past 123 and 131 years for temperature and precipitation, respectively. Simple linear regressions of temperature and precipitation were run separately for each month (24 total regressions).

To compare meteorological conditions during the modern observation period versus meteorological conditions during Mikesell’s historic observation period, we calculated temperature anomalies for each month in the modern observation years (2010–2014) relative to the average temperature of each month during the historic period, ${\bar{T}}_{i,hist}$. All data used to calculate temperature anomalies was taken from the USHCN. Monthly temperature anomalies for the modern period, *T*_*Aix*_, were calculated by subtracting the mean temperature for month *i* in modern collection year *x*, ${\bar{T}}_{ix}$, from the historic mean monthly temperature for month *i*, ${\bar{T}}_{i,hist}$ such that

$$T_{Aix}={\bar{T}}_{i,hist}‐{\bar{T}}_{ix}.$$

Thus, positive or negative anomalies indicate that a given month was warmer or cooler than in the historic period, respectively. ${\bar{T}}_{i,hist}$ is calculated as the mean of all temperatures for month *i* from 1892–1912 as follows

$${\bar{T}}_{i,hist}=\sum_{1892}^{1912}T_{i}/21.$$

Because USHCN temperature data is available beginning in 1892, *T*_*i*,*hist*_ does not include the first ten years of Mikesell’s observation period.

Monthly precipitation anomalies in modern observation years (2010–2013) relative to historic average monthly precipitation (cm) were calculated similarly using USHCNata. Monthly precipitation anomalies for the modern period, *P*_*Aix*_, were determined by subtracting the total precipitation for month *i* in modern collection year *x*, *P*_*ix*_, from the historic mean monthly total precipitation for month *i*, *P*_*i*,*hist*_ such that

$$P_{Aix}=P_{i,hist}‐P_{ix}.$$

Thus, positive or negative anomalies indicate that a given month received more or less precipitation than in the historic period, respectively. *P*_*i*,*hist*_ is calculated as the mean of all precipitation totals for month *i* from 1883–1912 as follows

$$P_{i,hist}=\sum_{1883}^{1912}P_{i}/30.$$

### III. Phenological observations

#### i. Historic observation period

Thomas Mikesell completed his historic phenological observations of 48 woody trees, shrubs, and vines from 1883–1912. Mikesell’s leaf phenology record is unique among historic datasets as he observed the timing of budburst in the spring as well as the timing of foliage coloration and leaf fall in the autumn allowing assessment of total growing season length. His leaf phenophase categories included “Buds start,” “Complete change of foliage,” and “Divested of leaves.” On average, Mikesell recorded budburst, foliage coloration, and leaf fall for the seven study species in 18 of the 30 historic observation years making his dataset one of the longest autumn phenology data sets published. Mikesell made observations multiple times weekly based on his collection records. However, it is unknown how many individuals per species Mikesell observed each year as he provided a single day of year (DOY, where 1 = Jan. 1 and 365 = Dec. 31) observation for each species and phenophase in each year.

#### ii. Modern observation period

Modern observations of leaf phenology for the seven study species were completed from 2010–2014 for a total of five and four spring and fall observation seasons, respectively. Observations were conducted in three plots located within 5 kilometers of Mikesell’s farmhouse and all species were represented by individuals in at least two of the three plots. All plots were privately owned by local residents and we gained land-owner permission to access each plot prior to observations. No additional permits were required and the sites were not altered in any way by our observations. All individuals were healthy, mature, and located in the plots’ interiors except for staghorn sumac which is a shrub species present only at plot edges. The total number of individuals observed varied between species ranging from thirteen (sassafras) to six (black walnut) and also varied among years as individuals that were damaged as a result of intense storms, Dutch Elm disease, or farm equipment were no longer observed.

We completed modern observations of spring and fall leaf phenology every 3–7 days with an average of 4.7 and 5.1 days between observations in the spring and fall, respectively. Budburst (equivalent of Mikesell’s “Buds start”) was defined as the first appearance of green leaf tissue from the buds occurring in at least 3 buds of an individual. Foliage coloration and leaf fall dates (equivalent of Mikesell’s “Complete change of foliage,” and “Divested of leaves”) were defined as the dates at which at least 90–95% of the leaves of an individual’s canopy were colored or had fallen, respectively. Growing season length was defined as the difference in days between the DOY of foliage coloration and the DOY of budburst. Foliage coloration date was selected as the end of the growing season as full canopy coloration more accurately approximates the end date of substantial photosynthetic activity than the point at which over 95% of leaves have fallen. Further, previous research indicates that observed increases in the duration of C sequestration resulted from a longer period of autumn photosynthetic activity [33] suggesting that our GSL definition more tightly links phenology with C exchange dynamics than would a leaf fall based GSL definition.

#### IV. Relationships with climatological variables

Mikesell’s meteorological data [30] was used to correlate mean monthly temperature and precipitation with historic phenophase DOYs as temperature data from the USHCN was not available for the first 10 years of the historic observation period. To determine significant relationships for each phenophase in each species, we calculated correlation coefficients for each species’ phenophase DOYs versus the mean monthly temperature and total monthly precipitation values for the average month of the phenophase and the 11 months prior (2 drivers x 12 months = 24 correlations per phenophase in each species). For example, budburst in black walnut occurred on May 7^th^ on average. Thus, black walnut budburst dates were correlated with mean monthly temperatures and total monthly precipitation values of January-May in the same year in which budburst occurred and June-December of the year prior. These correlation analyses were completed individually for each species and each phenophase (21 total correlation analyses). Correlation analyses were completed in JMP 16 statistical software.

#### V. Statistical analyses

In line with our objective to investigate species-specific differences in leaf phenological responsiveness, we assessed differences in the timing (DOY) of historic versus modern budburst, foliage coloration, leaf fall, and GSL for each of the seven study species individually using one-way ANOVAs (α = 0.05). Mikesell’s dataset included only one DOY for each species’ phenophase in a given year. Thus, to compare the historic yearly phenophase DOYs with modern phenophase dates, we calculated a yearly mean DOY for each species and each phenophase in the modern observation period. Species-specific yearly mean phenophase dates were calculated as follows:

$${\bar{DOY}}_{BB,sx}=\left(\sum_{i=1}^{n_{sx}}{DOY}_{BB,syx}\right)/n_{sx}$$

$${\bar{DOY}}_{FC,sx}=\left(\sum_{i=1}^{n_{sx}}{DOY}_{FC,syx}\right)/n_{sx}$$

$${\bar{DOY}}_{LF,sx}=\left(\sum_{i=1}^{n_{sx}}{DOY}_{LF,syx}\right)/n_{sx}$$

where *DOY_BB,syx_, DOY_FC,syx_*, and *DOY_LF,syx_* are the DOY of budburst, foliage coloration, and leaf fall, respectively, in individual *y* of species *s* in year *x*, and *n_sx_* is the number of individuals observed for species *s* in year *x*. Calculation of a yearly mean DOY for each species’ phenophase allows comparisons of yearly phenophase dates between the historical and modern time periods rather than comparing yearly dates (historical) with phenophase dates of several individuals within the same year (modern). ANOVAs comparing yearly modern and historic phenophase DOYs and GSL were completed in JMP Pro 16 statistical software [34].

## Results

### I. Long-term temperature and precipitation trends

**Fig 1:**
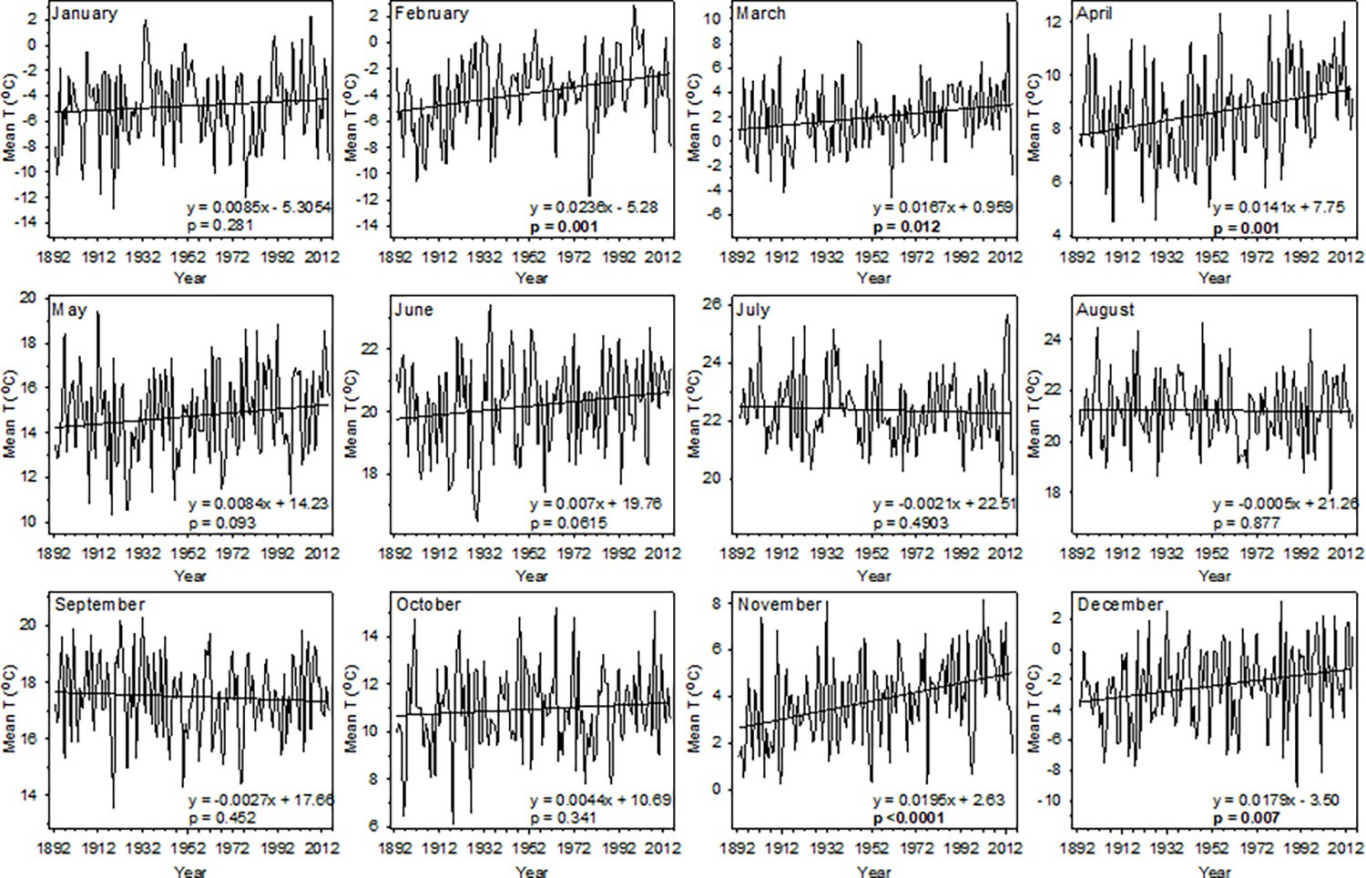
Trends in mean temperature for each month in Wauseon, OH from 1892–2014. Simple linear regressions were used to determine the rate of temperature change in each month (°C/year) over the 123-year period. Significant *p*-values are given in bold.

Wauseon has experienced significant warming in mean monthly temperatures over the past 123 years although this warming is concentrated in the mid-winter and spring (Fig 1). February, November, December, March, and April experienced, in descending order, the greatest warming ranging from 2.9 (0.24°C/decade) to 1.7°C (0.14°C/decade) warming since 1892 (*p* < 0.05, Fig 1). Further, four of the ten warmest years on record in January and February and six of the ten warmest years in November and December have occurred since 1990. The remaining months had no significant trends in temperature over time.

**Fig 2:**
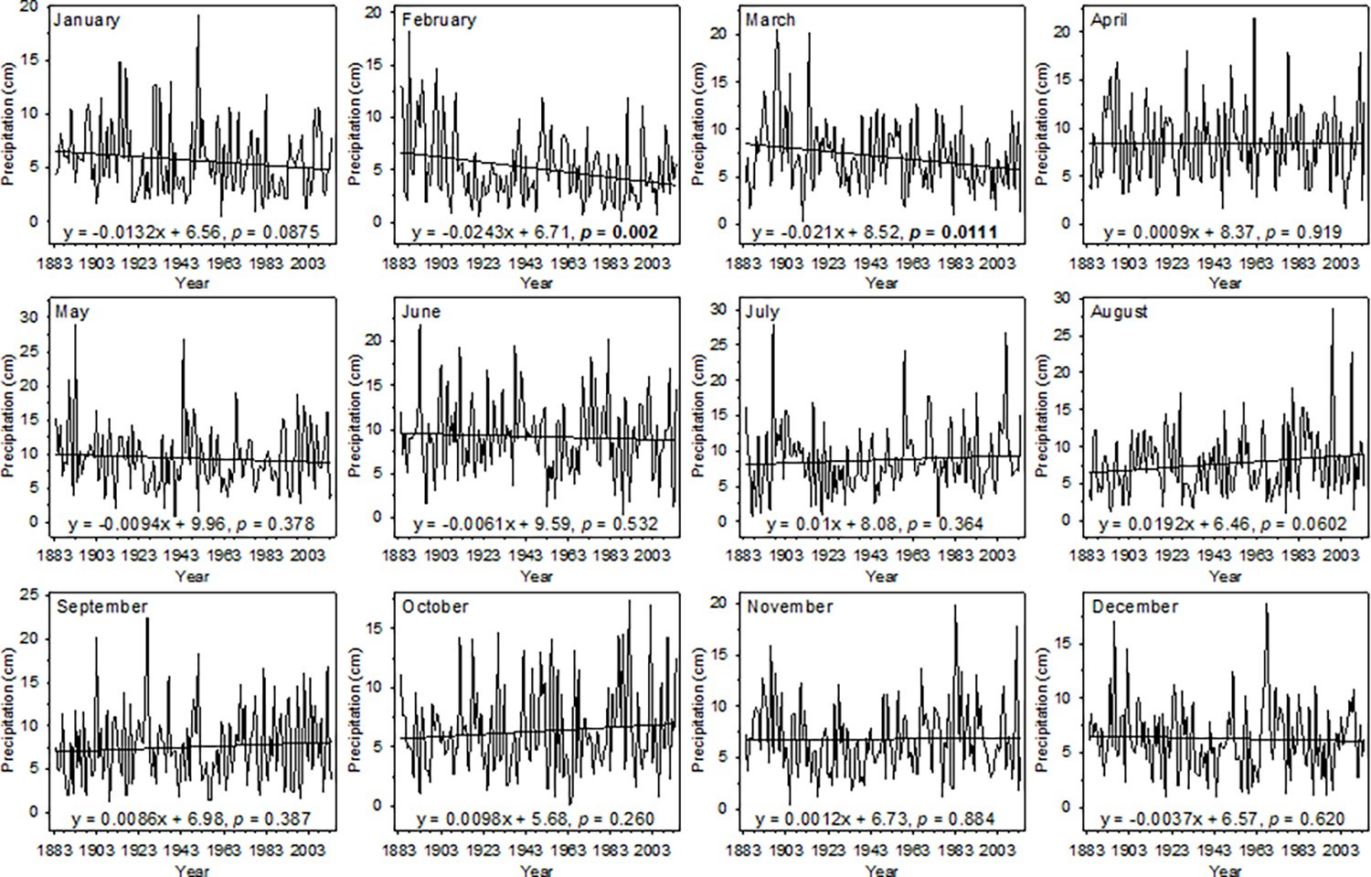
Trends in total precipitation for each month in Wauseon, OH from 1883–2013. Simple linear regressions were used to calculate the rate of change in precipitation amounts in each month (cm/year) over the 131-year period. Significant *p*-values are given in bold.

Total monthly precipitation was relatively stable over the 1883–2013 period with no significant trends in any months except February and March (Fig 2). Total monthly precipitation in February and March decreased significantly during the past 131 years by 3.2 (0.27cm/decade) and 2.8 cm (0.23cm/decade), respectively (*p* < 0.05, Fig 2).

### II. Meteorological conditions during the modern observation period

**Fig 3:**
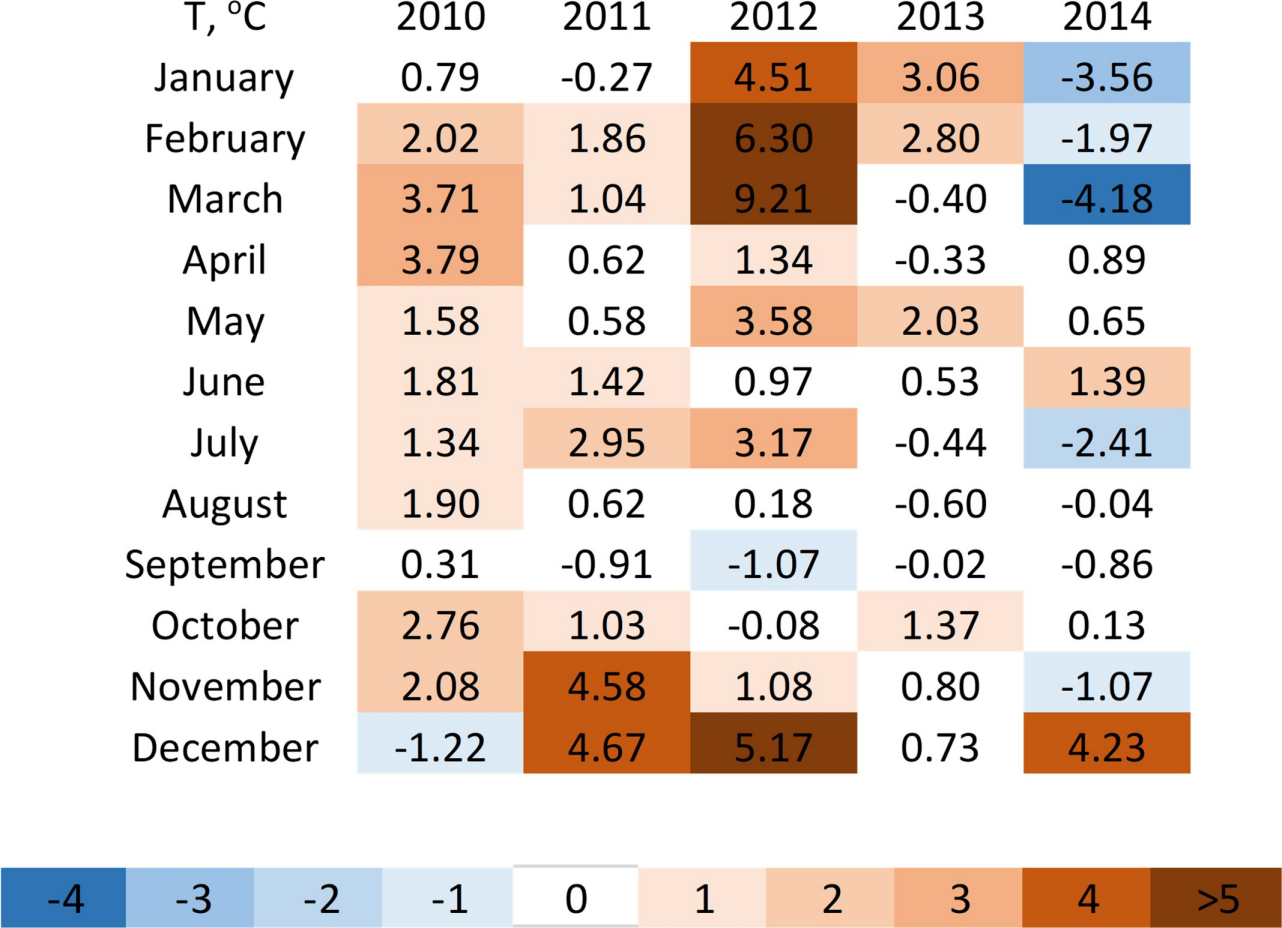
Monthly temperature anomalies (*T*_*Aix*_) in modern observation years (2010–2014) relative to historic average monthly temperatures (*T*_*i*,*hist*_, 1892–1912, °C). Positive or negative anomalies indicate that a given month was warmer or cooler than in the historic period, respectively.

Mean monthly temperatures varied substantially between years in the modern observation period (Figs 1 and 3) and thus the degree to which temperature conditions in the modern observation period were anomalous relative to the average temperature conditions during Mikesell’s historic observations also showed a high degree of variability (Fig 3). Further, the set of months that experienced the greatest warming relative to the historic period varied between years. The extremely warm winter to spring at the end of 2011 and beginning of 2012, respectively, is of particular note with a five-month period from November to March in which temperatures ranged from 4.5 to 9.2°C warmer than during the historic period. The March of 2012 was the hottest in the entire 123-year time series. In contrast, the beginning of 2014 was unusually cold with average temperatures from January to March ranging from 2 to 4.2°C colder than during the historic period making 2014 the fourth coldest March on record. However, it is important to note that 30 of the 60 months in the modern period experienced temperature anomalies that were at least 1°C warmer than the historic period while only 7 monthly had temperature anomalies that were 1°C cooler.

**Fig 4:**
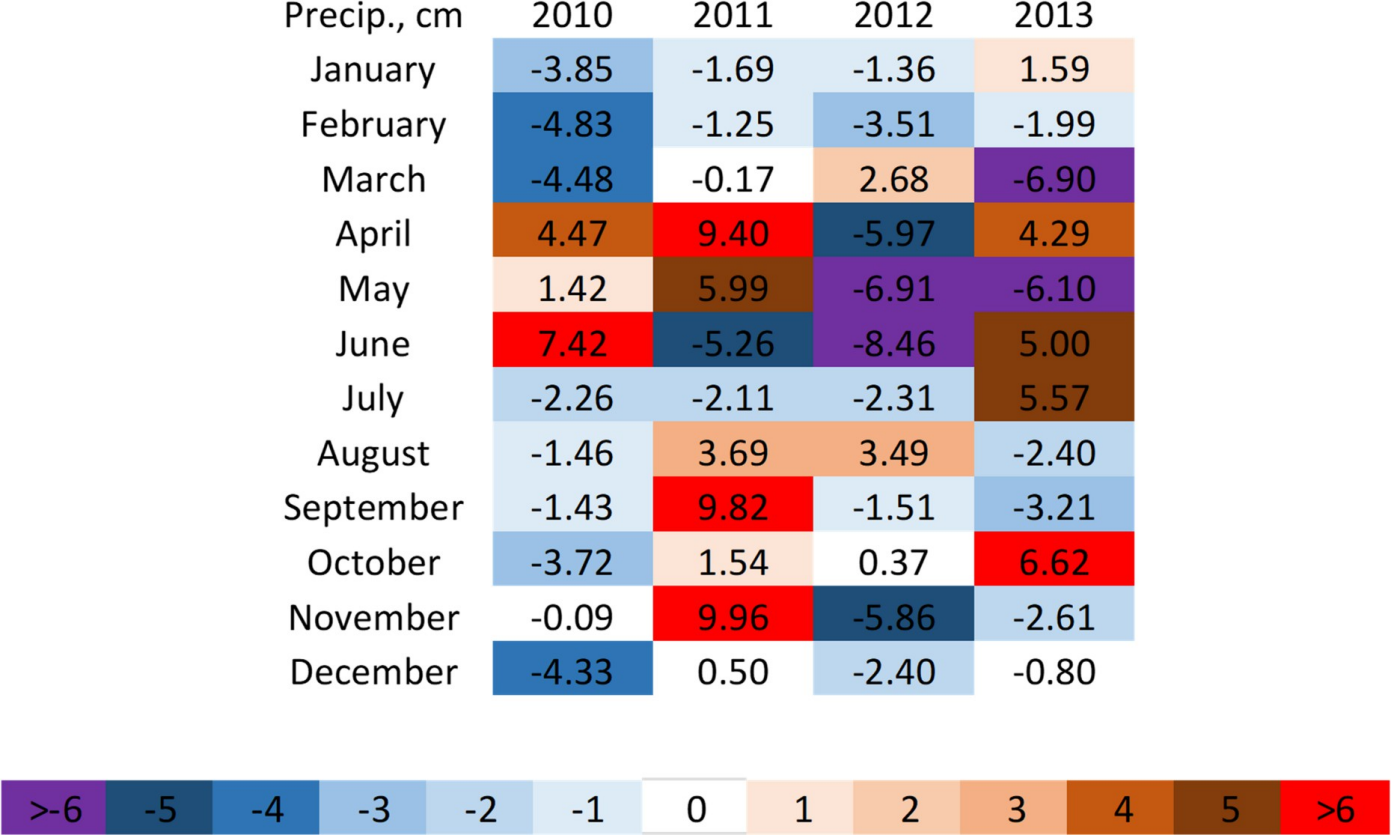
Monthly precipitation anomalies (*P*_*Aix*_,) in modern observation years (2010–2013) relative to historic average monthly total precipitation (*P*_*i*,*hist*_, 1883–1912, cm). Positive or negative anomalies indicate that a given month received more or less precipitation than in the historic period, respectively.

The modern observation period tended to be slightly drier than the historic period with 27 of 48 months experiencing precipitation anomalies that were at least 1 cm drier than the historic period. Only 16 months had precipitation anomalies that were 1cm wetter than during the historic period. However, total monthly precipitation anomalies in the modern period varied substantially between years. With the exception of February, no months were consistently wetter or drier than in the historic period in all four years (Fig 4).

### III. Historic vs. modern leaf phenology

#### i. Budburst

**Fig 5:**
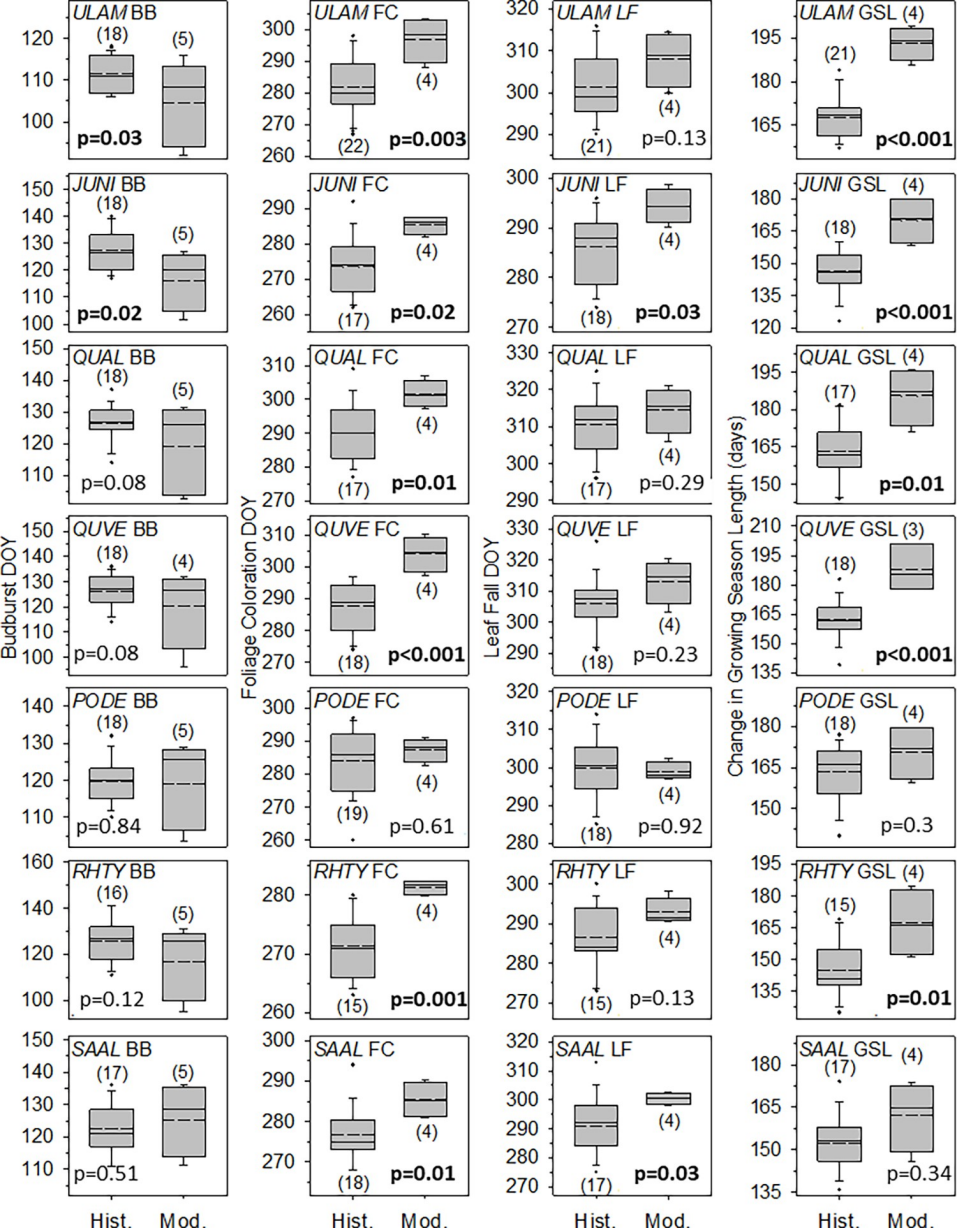
Comparison of historic versus modern timing of budburst, foliage coloration, and leaf fall, and growing season length for the seven focal species. Timing of each phenophase is given as the day of year (DOY) where 1 = Jan. 1 and 365 = Dec. 31. For each box, mean and median dates for each phenophase are indicated by dashed and solid lines, respectively, and outliers are indicated by points. Species abbreviations are *ULAM* = *U*. *americana*, *JUNI* = *J*. *nigra*, *QUAL* = *Q*. *alba*, *QUVE* = *Q*. *velutina*, *PODE* = *P*. *deltoides*, *RHTY* = *R*. *typhina*, and *SAAL* = *S*. *albidum*. Significant *p*-values are given in bold and sample sizes are given in parentheses.

Budburst showed relatively weak advancement in the modern period relative to the historic period. Only black walnut and American elm showed significantly earlier budburst in the modern period (12 and 8 days earlier, p = 0.021 and 0.028, respectively, Fig 5). In contrast, cottonwood, white oak, black oak, staghorn sumac, and sassafras showed no significant advances (p > 0.05, Fig 5). Generally, the average budburst DOY in the modern period was earlier than in the historic period for elm, black walnut, white oak, black oak, and sumac with black walnut and black oak showing the greatest and least advancement of budburst, respectively (10.9 and 5.8 days earlier, respectively, Fig 5, S1 Tables in S1 File). In contrast, Cottonwood showed no change in budburst DOY (0.7 days earlier on average) and budburst in Sassafras was somewhat delayed relative to the historic period (2.8 days later). Further, DOY of budburst within each of the seven focal species showed wide interannual variation in the modern observation period. On average, across the 5 years of the modern observation period, the DOY of budburst varied by roughly a month (28.6 days, Fig 5, S1 Tables in S1 File) with budburst DOY for Sumac and Black oak varying by roughly 36 days.

#### ii. Foliage coloration

Foliage coloration was significantly delayed in all focal species except cottonwood (p < 0.05, Fig 5). Foliage coloration was most delayed in black oak (16.2 days later in the modern period) and experienced the least delay in sassafras (8.6 days later, Fig 5, S2 Tables in S2 File). The DOY of foliage coloration also showed interannual variation, although to a lesser degree than budburst (S2 Tables in S2 File). Across the 7 species, the average interannual variation in foliage coloration DOY was 9.2 days with elm and sumac showing the greatest and least variability (15.6 and 2.5 days, respectively, Fig 5, S2 Tables in S2 File).

#### iii. Leaf fall

The leaf fall DOY was unchanged between the historic and modern periods except in Black walnut, Sumac, and Sassafras which delayed leaf fall by 8.1, 6.4, and 9.7 days, respectively (p < 0.05, Fig 5, S3 Tables in S3 File). Interannual variation in leaf fall DOY was comparable to that of foliage coloration with average variation of 10.4 days among all seven focal species. The greatest and least interannual variation in leaf fall DOY was experienced by black oak (17 days) and sassafras (4.4 days), respectively (Fig 5, S3 Tables in S3 File).

#### iv. Growing season length

Growing season length in Wauseon was 19.6 days longer, on average, in the modern observation period with five of the seven focal species experiencing significant growing season elongation (elm, black walnut, white oak, black oak, sumac Fig 5, S4 Tables in S4 File). Black oak and sumac showed the greatest (26 days) and least (22.3 days) growing season elongation, respectively, among those species with significantly longer growing seasons in the modern period. Growing season length in the modern period also showed substantial interannual variation with the length of the longest vs. shortest growing season differing by 33.5 days (sumac) to 13.4 days (elm).

### IV. Association with climatological variables

#### i. Budburst

Generally, mean temperatures in the month of and the month immediately preceding budburst (April and May) were significant predictors of budburst with warmer temperatures resulting in earlier timing of budburst (Table 1). April and May mean temperatures were significantly related to budburst DOY in white oak, black oak, and staghorn sumac while sassafras and black walnut were significantly associated with only mean April temperature. Warmer temperatures were associated with earlier timing of budburst for all significant mean monthly temperature variables and in all species. Mean monthly temperatures in the winter months preceding budburst were also significantly correlated for black walnut (January and November mean temperatures) and sassafras (January mean temperatures, Table 1). Cottonwood budburst was not significantly correlated with any temperature variable (Table 1). Of the 84 correlations between monthly temperatures and budburst DOY, 69% were negative indicating an average shift to earlier budburst timing with warmer conditions. When considering only spring and winter temperature predictors (December of the preceding year and January-May preceding budburst), 79% of all correlations indicated a negative relationship between temperature and budburst timing.

**Table 1:** Correlation coefficients for budburst DOY with mean monthly temperature (oC) and total monthly precipitation (cm) for the month of and 11 months preceding budburst in the seven focal species.

	*J*. *nigra*	*P*. *deltoides*	*Q*. *alba*	*Q*. *velutina*	*R*. *typhina*	*S*. *albidum*	*U*. *americana*
Jan. T	**-0.624** **	0.233	-0.352	-0.420	-0.301	**-0.650**	0.210
Feb. T	-0.130	0.294	-0.106	-0.222	-0.181	-0.395	-0.280
Mar. T	-0.387	0.207	-0.074	-0.163	-0.130	-0.436	0.242
Apr. T	**-0.646** **	-0.374	**-0.844** ***	**-0.901** ***	**-0.759** ***	**-0.731** ***	0.102
May T	-0.251	-0.041	**-0.481** *	**-0.591** **	**-0.551** *	-0.412	0.265
Jun. T	-0.144	-0.446	-0.114	-0.127	-0.272	-0.064	0.336
Jul. T	-0.242	-0.095	-0.350	-0.363	-0.180	-0.150	0.452
Aug. T	-0.104	0.021	0.009	-0.091	-0.173	-0.107	0.279
Sep. T	0.269	-0.258	0.164	0.108	0.105	-0.073	0.387
Oct. T	0.358	-0.062	0.239	0.172	0.189	0.189	0.418
Nov. T	**-0.594** *	0.051	-0.314	-0.209	-0.381	-0.370	-0.011
Dec. T	-0.306	0.245	0.000	-0.029	-0.066	-0.483	0.007
Jan. P	-0.160	0.167	-0.199	-0.188	0.012	-0.237	0.216
Feb. P	0.276	0.249	0.156	0.064	0.068	0.200	-0.394
Mar. P	-0.198	0.135	0.110	0.063	-0.040	-0.337	0.435
Apr. P	-0.034	-0.116	-0.009	-0.062	-0.025	-0.294	-0.220
May P	0.082	0.247	0.461	**0.491** *	0.291	0.057	**-0.543** *
Jun. P	0.152	0.116	0.313	0.340	0.249	0.145	-0.174
Jul. P	0.019	-0.023	0.065	0.152	0.218	0.097	0.174
Aug. P	0.020	-0.095	-0.188	-0.240	-0.423	-0.173	-0.415
Sep. P	0.301	-0.037	-0.015	-0.096	-0.127	0.343	-0.110
Oct. P	-0.129	-0.419	-0.258	-0.211	-0.204	0.067	0.166
Nov. P	-0.243	-0.007	0.043	0.008	-0.070	-0.461	0.019
Dec. P	-0.150	-0.405	-0.332	-0.356	-0.122	-0.207	0.028

Bolded values are significant.

*denotes p ≤ 0.05

**denotes p ≤ 0.01

***denotes p ≤ 0.001

Total May precipitation was significantly correlated with budburst in black oak and elm while no other precipitation values were significantly related to budburst in any other species. The effects of increasing precipitation were opposite in these species with wetter Mays driving delayed and earlier budburst in black oak and elm, respectively (Table 1).

#### ii. Foliage coloration

Foliage coloration was significantly correlated with at least one mean temperature term for all species except for black walnut, sassafras, and elm (Table 2) and in all cases, warmer temperatures in the predictor month resulted in delayed timing of foliage coloration. Generally, significant temperature variables were from the winter and spring preceding foliage coloration. Mean December temperatures in the previous year were significantly correlated with foliage coloration for white oak, black oak, and cottonwood. Sumac foliage coloration was significantly correlated with April and August mean temperatures. No monthly temperature variables were significantly correlated with foliage coloration in black walnut, sassafras, or elm. Across the 84 correlations of monthly temperature variables with foliage coloration date, 54% were positive (indicating delayed foliage coloration with warming).

**Table 2:** Correlation coefficients for foliage coloration DOY with mean monthly temperature (°C) and total monthly precipitation (cm) for the month of and 11 months preceding budburst in the seven focal species.

	*J*. *nigra*	*P*. *deltoides*	*Q*. *alba*	*Q*. *velutina*	*R*. *typhina*	*S*. *albidum*	*U*. *americana*
Jan. T	-0.246	0.409	0.044	0.161	0.056	-0.075	0.058
Feb. T	-0.093	0.191	-0.165	0.144	0.019	-0.144	-0.209
Mar. T	-0.333	0.234	0.069	0.059	-0.158	-0.103	0.349
Apr. T	0.120	0.081	-0.087	0.010	**0.627** *	-0.049	-0.138
May T	-0.239	-0.076	-0.104	-0.136	0.425	-0.074	-0.225
Jun. T	0.234	0.242	-0.147	0.154	0.264	-0.180	-0.146
Jul. T	-0.024	0.311	-0.041	-0.154	-0.096	-0.055	-0.164
Aug. T	0.087	0.235	-0.028	0.088	**0.576** *	-0.141	0.317
Sep. T	0.047	0.280	0.135	-0.130	-0.052	0.288	0.067
Oct. T	0.274	0.175	0.044	-0.129	0.008	0.317	0.133
Nov. T	-0.391	-0.189	-0.073	-0.144	-0.354	-0.206	0.074
Dec. T	0.145	**0.541** *	**0.531** *	**0.633** **	0.181	0.241	-0.117
Jan. P	0.214	0.227	0.052	-0.008	-0.165	-0.091	0.243
Feb. P	-0.110	-0.091	-0.340	-0.102	-0.237	-0.190	-0.391
Mar. P	0.122	0.321	-0.024	0.060	-0.189	0.077	0.037
Apr. P	0.389	0.208	-0.429	-0.290	0.474	-0.022	0.175
May P	-0.206	-0.017	-0.169	0.112	-0.057	0.051	-0.363
Jun. P	-0.183	-0.115	-0.454	-0.256	0.155	-0.143	-0.164
Jul. P	-0.216	-0.317	-0.373	-0.391	0.180	-0.225	-0.029
Aug. P	0.180	0.154	0.106	-0.036	**0.640** **	**0.475** *	0.213
Sep. P	-0.033	0.093	0.059	0.238	**0.647** **	-0.034	0.410
Oct. P	-0.212	-0.170	0.113	0.054	-0.158	-0.094	-0.038
Nov. P	-0.012	0.086	0.085	0.410	0.224	-0.100	0.074
Dec. P	-0.270	-0.070	-0.157	-0.130	0.308	-0.088	-0.074

Bolded values are significant.

*denotes p ≤ 0.05

**denotes p ≤ 0.01

***denotes p ≤ 0.001

Total monthly precipitation in at least one month was a significant predictor of foliage coloration only in sumac and sassafras (Table 2). Increased precipitation in August was associated with later timing of foliage coloration in both species and wetter Septembers were also associated with later foliage coloration in sumac (Table 2).

#### iii. Leaf fall

For all mean monthly temperature terms, higher temperatures resulted in earlier timing of leaf fall except for September and October temperatures which were only significantly correlated with cottonwood leaf fall (Table 3). Other than mean January temperatures, which were significantly related to leaf fall for elm, black walnut, sumac, and sassafras, there was little consistency between species regarding which monthly temperatures were predictive of leaf fall. (Table 3). In contrast with foliage coloration, a majority of correlations (69%) with mean monthly temperatures were negative (indicating warmer temperatures are associated with earlier leaf fall).

**Table 3:** Correlation coefficients for leaf fall DOY with mean monthly temperature (°C) and total monthly precipitation (cm) for the month of and 11 months preceding budburst in the seven focal species.

	*J*. *nigra*	*P*. *deltoides*	*Q*. *alba*	*Q*. *velutina*	*R*. *typhina*	*S*. *albidum*	*U*. *americana*
Jan. T	**-0.487** *	-0.036	-0.426	-0.329	**-0.629** *	-0.638**	**-0.453** *
Feb. T	-0.426	0.061	-0.116	0.077	-0.392	-0.132	-0.137
Mar. T	-0.300	0.221	-0.342	-0.266	-0.388	-0.470	0.034
Apr. T	-0.149	-0.148	-0.259	-0.220	-0.204	**-0.527** *	-0.286
May T	-0.285	-0.291	-0.363	-0.328	0.102	-0.415	0.014
Jun. T	0.124	0.124	-0.146	0.251	-0.071	-0.304	-0.321
Jul. T	-0.317	0.176	-0.403	-0.437	-0.122	-0.391	-0.036
Aug. T	-0.119	-0.044	-0.481	-0.240	-0.029	**-0.578** *	-0.257
Sep. T	0.110	**0.608** **	0.110	0.005	-0.099	-0.054	0.160
Oct. T	0.145	**0.669** **	0.297	0.027	0.012	0.201	0.372
Nov. T	-0.255	0.060	-0.042	-0.443	-0.444	-0.049	0.096
Dec. T	-0.041	0.169	-0.224	-0.407	-0.337	-0.146	-0.314
Jan. P	-0.360	0.202	0.232	-0.115	-0.328	-0.101	0.173
Feb. P	0.001	0.056	0.144	0.340	-0.102	0.165	0.131
Mar. P	-0.248	0.345	-0.146	-0.098	-0.511	-0.177	-0.139
Apr. P	-0.087	0.195	-0.215	-0.223	-0.029	-0.378	-0.276
May P	0.136	-0.337	**-0.493** *	0.069	0.044	-0.023	-0.414
Jun. P	0.082	-0.215	-0.384	-0.094	0.223	-0.034	-0.388
Jul. P	-0.234	-0.322	-0.196	-0.270	0.060	-0.124	-0.129
Aug. P	0.016	-0.102	-0.030	-0.190	0.405	0.032	0.104
Sep. P	-0.073	0.001	-0.412	0.022	0.424	-0.011	0.192
Oct. P	0.372	0.211	**0.657** **	0.431	0.271	**0.506** *	0.243
Nov. P	-0.448	-0.249	-0.096	-0.129	-0.199	-0.228	-0.131
Dec. P	-0.346	-0.243	-0.425	-0.356	0.165	-0.396	-0.203

Bolded values are significant.

*denotes p ≤ 0.05

**denotes p ≤ 0.01

***denotes p ≤ 0.001

Total monthly precipitation was significantly related with leaf fall only in white oak and sassafras. Leaf fall DOY in both species was significantly related with October precipitation with delayed timing in wetter years. In contrast, higher May precipitation was significantly correlated with earlier leaf fall DOY in white oak (Table 3).

## Discussion

Our study indicates that climate change is already altering the timing of key seasonal events in forests of eastern North America as the growing season has been significantly extended over the past 130 years in five of the seven study species. This growing season elongation occurred because of delayed foliage coloration and, less consistently, due to advanced timing of budburst. The magnitude of shifts in the timing of both spring and fall leaf phenological events were variable among the seven species. Further, budburst, foliage coloration, and leaf fall were significantly related to different monthly temperature and precipitation variables suggesting that variation in leaf phenology results from interspecific differences in the drivers of each phenophase and sensitivity to those drivers. Thus, this research confirms that species-specific assessments of shifting leaf phenology are necessary to accurately predict the consequences of climate change in mixed-species temperate deciduous forests.

### I. Historic vs. modern phenology

#### i. Budburst

In contrast with previous research, we found weaker trends for budburst advancement than for autumn foliage coloration delays [1, 2, 6, 35, 36] with only two of the seven focal species displaying advanced timing of budburst in the modern period. However, the apparent lack of budburst advancement over the past century in the majority of our study species was likely due to limited sampling years in the modern period and substantial interannual variability in spring temperature (Fig 3). Spring temperatures in two of the five modern observation years, 2014 and 2011, were colder than or comparable to the average temperatures during Mikesell’s observations, respectively. As a result, budburst was not advanced relative to the historic period in either of these years (S1 Tables in S1 File). In contrast, the warm springs of 2010 and 2012 resulted in average advancement of budburst across all seven species of roughly 19 days with both sumac and black walnut experiencing budburst a month earlier in 2012 than the average historic timing (S1 Tables in S1 File). Given the consistent warming trend over the past 120 years in February, March, and April (Fig 1) and the strong advancement of budburst observed in warm years, spring leaf phenology in Wauseon has likely advanced substantially over the past century.

Further, the substantial interannual variation observed in the timing of budburst may become increasingly common as climate change is associated with more extreme variation in weather [37]. Over the five observation years, the timing of budburst varied by roughly a month on average for all seven species (S1 Tables in S1 File) and this variation is consistent with previous research demonstrating greater interannual differences in the timing of spring than in autumn phenological events [38]. The considerable interannual variability in budburst has significant implications for annual ecosystem carbon storage calculations and the development of trophic mismatches for organisms that rely on food sources associated with spring leaf out [21, 39]. Further long-term observations are required to quantify changes in the degree of interannual variation in budburst timing with climate change and to assess its ecological effects.

#### ii. Foliage coloration and leaf fall

Changes in the timing of foliage coloration and leaf fall varied among species with a trend toward delayed timing of coloration while leaf fall remained relatively stable. These differences in the shift of coloration and leaf fall dates emphasize the importance of monitoring individual phenophases as each event responds to a unique set of climate and endogenous drivers [1, 18, 40]. While six species experienced significant delays in foliage coloration, leaf fall was delayed only in black walnut, sumac, and sassafras with weaker delays in leaf fall than foliage coloration in both walnut and sumac.

The greater delay in foliage coloration than in leaf fall suggests an overall compression of the duration of complete canopy coloration (defined as the period of during which the majority of the canopy, >90%, is colored) which may positively or negatively impact nutrient remobilization efficiency. Nitrogen (N) remobilized during senescence is particularly important as it provides almost all N required for budburst in the spring. Incomplete or inefficient resorption can limit the advancement of budburst with favorable spring temperatures and thereby limit gains in C sequestration [41, 42]. A handful of studies have assessed the effects of timing *versus* duration of leaf senescence on N remobilization efficiency [43]. For example, later timing of leaf senescence resulted in greater N resorption in stands of both birch (*Betula pendula*) and beech (*Fagus sylvatica*, [44]). However, this increase in N remobilization has not been causally linked to delayed senescence and instead may reflect diminished remobilization efficiency in leaves that senesced prematurely due to environmental stressors like drought [45] or herbivory [43, 46, 47]. Alternatively, others have hypothesized that a longer duration of leaf senescence rather than delayed timing of senescence may increase N remobilization by providing a longer period for the numerous and varied physiological processes required for nutrient resorption [43]. This hypothesis suggests the observed compression of the duration of complete canopy coloration may negatively impact N resorption. However, if the onset of foliage coloration remains stable or is delayed less than full canopy coloration, the total period of leaf senescence may be extended although the duration of full coloration is compressed. Given the potentially significant effects of delayed leaf senescence and N remobilization efficiency, future research should investigate the duration of foliage coloration from the first observable loss of chlorophyll to total coloration, the duration of total canopy coloration, and their relationships to N resorption.

#### iii. Growing season length

In contrast with previous research, the delay in autumn foliage coloration, on average, contributed equally or more to total growing season elongation than advancement of budburst [2, 6, 35, 36] although the relative contribution of shifting budburst or foliage coloration to growing season elongation varied considerably among species. For example, black walnut and sumac gained roughly 10 days at the beginning and end of the growing season as budburst and foliage coloration shifted equally in these species. In contrast, delayed foliage coloration extended the growing season 2 to 3-fold more than earlier budburst in elm, black oak, and sassafras. As majority of studies only assess changes in the beginning of the growing season, this research suggests that estimates of climate change impacts on C sequestration and other important ecosystem processes may be substantially skewed because of inadequate data regarding species-specific changes in the end of the growing season.

### II. Relationships with climatological variables

#### i. Budburst

Temperature is considered the most important driver of budburst in temperate deciduous ecosystems and has a dual role in triggering budburst: chilling and thermal accumulation [21]. Cold temperatures during the “chilling period” in the late winter and early spring are required to release dormancy. Until chilling requirements are met, dormant buds are unable to respond to heat (thermal) accumulation which drives the physiological processes required to burst bud in the spring [24, 48]. In accordance with previous research, we found significant relationships between the timing of budburst and at least one mean monthly temperature in models constructed for all study species except cottonwood and elm. However, these mean monthly temperatures likely affect budburst timing via different mechanisms based on the stage of bud development during that season.

As in previous studies, increased temperatures in the month of or the month immediately preceding budburst was significantly associated with advanced budburst for five of the seven study species [15, 21, 49, 50]. Budburst was strongly correlated with warmer April temperatures for all five species. The inclusion of winter temperature variables in budburst models for both black walnut (mean January and November temperatures) and sassafras (mean January temperatures) suggested that chilling, along with thermal accumulation, is important in driving budburst for these species. The required amount of chilling varies among species as does the optimal range of chilling temperatures. Generally, temperatures below or above 0 and 10°C, respectively, are considered ineffective in meeting chilling requirements [15, 24, 51, 52]. Further, as buds are exposed to increased chilling time, the amount of warm, forcing temperatures (thermal time) required for budburst decreases although the relationship between chilling and thermal time required for budburst again varies substantially among species [53]. Extremely cold temperatures (below 0°C) in,January, resulted in significantly later budburst in black walnut and sassafras likely as a result of late fulfillment of each species’ chilling requirements. Similarly, previous research found that budburst in walnut and sassafras was best predicted by models that included both chilling requirements and thermal accumulation [31]. with sassafras budburst also strongly dependent on high chilling requirements [54]. Given the significant warming trends for November, climate change is likely resulting in earlier fulfillment of chilling requirements for black walnut as temperatures above 0°C occur more frequently.

Precipitation is generally regarded as an insignificant driver of temperate broadleaf spring phenology and our models confirmed the greater relationship of temperature with budburst as significant correlations with temperature were four-times more common than with precipitation variables. Water availability primarily affects the start of the growing season in ecosystems with seasonal droughts like dry grasslands [55] and Mediterranean ecosystems [25] with summer droughts resulting in advanced budburst to allow completion of growth before the onset of substantial water stress [56]. Elm budburst was significantly related to precipitation in the previous spring. However, these results are not consistent with the drought responses of arid environments as increasing precipitation was associated with earlier timing of budburst. In contrast, increased precipitation in the month of budburst (May) was associated with delayed budburst in black oak. However, May temperature and precipitation are weakly, negatively correlated as cooler Mays were also generally wetter and thus the significant relationship between budburst date and May precipitation may be conflated with temperature.

#### ii. Foliage coloration

Identifying drivers of autumn leaf phenology has been challenging for a variety of reasons. The lack of long-term autumn phenology observations prevents robust assessment of climatological effects on autumn phenophases both among species and locations [57]. Further, distinct effects of temperature on foliage coloration between species are commonly reported [58]. Within species, individuals have been shown to have differing temperature sensitivities at different sites ranging from no temperature response to significantly delayed leaf fall [59]. The onset of responsiveness to cooling temperatures also varies among different ecotypes of the same species as their sensitivity to photoperiod cues varies with latitude [60].

Among the very few species-specific studies of autumn leaf phenology, most focus exclusively on temperature effects and ignore possible precipitation effects. While leaf fall in response to water stress in drought adapted species has been demonstrated [61], the effects of water availability on winter-deciduous species in temperate, non-drought adapted species has received little attention [62]. Furthermore, despite the lack of consensus on drivers of autumn phenology [15, 40], many studies restrict their analyses of drivers of autumn phenology to include temperature and precipitation variables for a limited period of the year, usually the period of leaf on [18, 63] (but see [16, 64, 65] for studies of lagged effects of previous seasons). The correlation analysis of this study suggests that discounting precipitation drivers and limiting our analyses to climatological variables during the growing season may significantly hinder both our predictive power for autumn leaf phenology and our understanding of the possible species-specific physiological underpinnings of senescence.

In accordance with previous research, warmer temperatures were related to delayed foliage coloration [18, 64, 66]. However, lagged temperature effects from the preceding spring and winter were significantly related to foliage coloration more frequently than temperatures from the months immediately preceding foliage coloration. These results differed from the few studies that have assessed lagged temperature effects on senescence which found significant relationships only with temperatures in the summer and autumn months immediately preceding foliage coloration [16, 64]. However, a winter warming experiment in oak and beech showed lagged winter temperature effects with increased winter temperatures leading to advanced leaf out and, in contrast with our results, advanced leaf senescence [65]. This earlier leaf senescence was hypothesized to be a consequence of budburst advancement resulting in earlier fulfilment of maximum sugar storage in sink organs rather than being driven directly by winter weather conditions [65]. As timing of budburst was not significantly correlated with foliage coloration or leaf fall for any of the seven species in this study and winter warming delayed rather than advanced foliage coloration, our results suggest a different mechanistic link between winter and spring temperatures and timing of foliage coloration.

Finally, increased precipitation in one or both of the months preceding foliage coloration (August and September) was associated with delayed senescence for two of the seven study species (sumac and sassafras). Similarly, other studies have found positive correlations between October precipitation and foliage coloration for both white and black oak although they hypothesize that this precipitation response may be conflated with temperature as these terms covaried in their analyses [18]. However, neither August nor September precipitation was significantly correlated with temperature in our analysis suggesting an independent role of precipitation in driving foliage coloration. This relationship is consistent with the hypotheses that increasing water limitation over the summer drives earlier onset of leaf senescence [16].

#### iii. Leaf fall

Surprisingly, the relationship between autumn precipitation variables and leaf fall was the opposite of the relationship between those variables and foliage coloration for those species with significant relationships with these variables. Wetter Octobers were linked to later leaf abscission for sassafras and white oak. In contrast, wetter Mays were associated with earlier leaf fall for white oaks. Similarly, while warmer temperatures were typically associated with delayed leaf coloration, they were associated with advanced leaf fall (54% of correlations with temperature predictors were positive for foliage coloration while 69% were negative for leaf fall).

We propose that the distinct effects of these variables on leaf fall compared with foliage coloration were not driven by differences in phenophase responses to climatological drivers. Instead, we hypothesize that these differences were likely due to constraints on the timing of leaf fall as a result endogenous factors such as leaf nutrient or non-structural carbohydrate content which are dependent on the timing of foliage coloration [40]. For all species except sumac and sassafras, the date of full canopy coloration was negatively associated with the duration of complete canopy coloration (defined as the number of days between full canopy coloration and complete leaf loss). That is, in years with earlier foliage coloration, the duration of complete canopy coloration was longer and accordingly, leaf fall occurred later. Oppositely, in years in which foliage coloration occurred later, the duration of complete canopy coloration was compressed and leaf fall occurred earlier. Thus, the climatological factors that drive advancement or delays in foliage coloration appear to cause later or earlier leaf fall, respectively.

We hypothesize that the relationship between the timing of foliage coloration and leaf fall is based on the duration of nutrient remobilization preceding full canopy coloration which may affect N remobilization and therefore the optimal timing of leaf abscission. If delays in full canopy coloration are also associated with a longer progression of coloration (defined as the total period of time from the first colored leaves to full canopy coloration), this may allow more time for nutrient resorption. Thus, nutrient remobilization would be more complete by the date of full canopy coloration and the leaves could abscise quickly thereafter resulting in relatively advanced leaf fall. In contrast, the progression of coloration likely would be briefer with earlier full canopy coloration. The shorter progression of coloration would lead to less complete nutrient resorption by the date of full canopy coloration. Thus, the extended duration of complete canopy coloration and subsequent delay in leaf fall allows additional N remobilization. The relationship between foliage coloration and fall was also consistent with the observed compression of the duration of complete canopy coloration in the modern observation period as a result of delayed foliage coloration while the timing of leaf fall remained relatively constant. To our knowledge, this relationship has not been reported previously in either observational or modeling studies.

## Conclusions

Our research confirmed the significant effects of climate change on growing season extension observed worldwide and added to the very limited number of studies on lagged effects of temperature from previous winters on budburst, the role of precipitation in both spring and fall leaf phenology, and the possible role of endogenous factors associated with foliage coloration and the timing of leaf fall. The findings regarding variables associated with autumn leaf phenology bear further investigation as the end of the growing season is particularly poorly understood. Thus, we suggest future research quantifying both the duration and timing of foliage coloration, the relationship between onset of coloration and nutrient remobilization efficiency, and the timing of leaf fall to better understand this critical event.

## Supporting information

S1 File Comparisons of modern versus historic budburst. Yearly budburst DOY observations for the seven focal species in the modern and historic observation periods, Species-specific average budburst DOY for the modern and historic observation periods, and Yearly variation in budburst DOY in the modern observation period.
(PDF)

S2 File Comparisons of modern versus historic foliage coloration. Yearly foliage coloration DOY observations for the seven focal species in the modern and historic observation periods, Species-specific average foliage coloration DOY for the modern and historic observation periods, and Yearly variation in foliage coloration DOY in the modern observation period.
(PDF)

S3 File Comparisons of modern versus historic leaf fall. Yearly leaf fall DOY observations for the seven focal species in the modern and historic observation periods, Species-specific average leaf fall DOY for the modern and historic observation periods, and Yearly variation in leaf fall DOY in the modern observation period.
(PDF)

S4 File Comparisons of modern versus historic growing season length (GSL). Yearly growing season lengths (GSL, days) for the seven focal species in the modern and historic observation periods, Species-specific average growing season length (days) for the modern and historic observation periods, and Yearly variation in total GSL in the modern observation period.
(PDF)
