# Supplementary material for: A century of climate warming results in growing season extension: Delayed autumn leaf phenology in north central North America
Source: PLoS One. 2023 Mar 3;18(3):e0282635. doi: 10.1371/journal.pone.0282635 (PMC9983848; doi:10.1371/journal.pone.0282635)
Supplement: S1 File — Yearly budburst DOY observations for the seven focal species in the modern and historic observation periods, Species-specific average budburst DOY for the modern and historic observation periods, and Yearly variation in budburst DOY in the modern observation period. (PDF) [file pone.0282635.s001.pdf]

**S1 File. Comparisons of modern versus historic budburst.** This file contains yearly and average values for historical and modern observations and interannual variation in budburst DOY among species in the modern period.

**S1 Table 1. Yearly budburst DOY observations for the seven focal species in the modern and historic observation periods**. Species abbreviations are *ULAM = U. americana*, *JUNI = J. nigra*, *QUAL = Q. alba*, *QUVE = Q. velutina*, *PODE = P. deltoides*, *RHTY = R. typhina*, and *SAAL = S. albidum.*

| Year | Observation Period | *ULAM* BB | *QUAL* BB | *JUNI* BB | *RHTY* BB | *PODE* BB | *SAAL* BB | *QUVE* BB |
|---|---|---|---|---|---|---|---|---|
| 2010 | Modern | 96 | 104.9 | 108.3 | 104.8 | 103.7 | 116.6 | |
| 2011 | Modern | 116 | 129.9 | 127.2 | 131 | 127.7 | 136 | 128.2 |
| 2012 | Modern | 92 | 102.9 | 101.7 | 95.1 | 109.4 | 111.4 | 96 |
| 2013 | Modern | 110.8 | 126 | 124.3 | 126 | 125.5 | 128.5 | 124.9 |
| 2014 | Modern | 108.4 | 131.6 | 120 | 126.8 | 129 | 134.3 | 131.9 |
| 1883 | Historic | 107 | 128 | 132 | | 115 | 130 | 133 |
| 1884 | Historic | 108 | 127 | 127 | 126 | 120 | 127 | 128 |
| 1885 | Historic | 113 | 133 | 139 | 141 | 114 | 136 | 136 |
| 1886 | Historic | 106 | 114 | 118 | 113 | 112 | 111 | 114 |
| 1887 | Historic | 106 | 130 | 140 | 128 | 129 | 132 | 125 |
| 1888 | Historic | 116 | 132 | 133 | 141 | 124 | 134 | 132 |
| 1889 | Historic | 111 | 127 | 120 | 121 | 128 | 119 | 128 |
| 1890 | Historic | 106 | 126 | 125 | 132 | 132 | 117 | 124 |
| 1891 | Historic | 111 | 120 | 120 | 113 | 115 | 118 | 119 |
| 1892 | Historic | 111 | 137 | 135 | 133 | 122 | 121 | 135 |
| 1893 | Historic | 106 | 133 | 134 | 132 | 123 | 126 | 132 |
| 1894 | Historic | 117 | 126 | 123 | 132 | 117 | 120 | 123 |
| 1895 | Historic | 116 | 126 | 130 | 122 | 121 | 124 | 123 |
| 1896 | Historic | 110 | 117 | 117 | 111 | 110 | 111 | 116 |
| 1897 | Historic | 114 | 127 | 126 | 124 | 120 | 124 | 127 |
| 1898 | Historic | 118 | 130 | 126 | 128 | 121 | 117 | 130 |
| 1899 | Historic | 116 | 118 | 118 | 117 | 117 | 116 | 117 |
| 1912 | Historic | 114 | 128 | 127 | | 117 | | 127 |

**S1 Table 2. Species-specific average budburst DOY for the modern and historic observation periods.** The change in average DOY budburst is calculated as the Modern average BB DOY - the Historic average BB DOY for each species.

| Observation Period | Average *ULAM* BB | Average *QUAL* BB | Average *JUNI* BB | Average *RHTY* BB | Average *PODE* BB | Average *SAAL* BB | Average *QUVE* BB |
|---|---|---|---|---|---|---|---|
| Modern | 104.6 | 119.1 | 116.3 | 116.7 | 119.1 | 125.3 | 120.3 |
| Historic | 111.4 | 126.6 | 127.2 | 125.9 | 119.8 | 122.5 | 126.1 |
| Change in average DOY BB | -6.8 | -7.5 | -10.9 | -9.2 | -0.7 | 2.8 | -5.8 |

**S1 Table 3. Yearly variation in budburst DOY in the modern observation period.** Variation in BB DOY between years is calculated as the difference between the latest and earliest DOY budburst for each species.

| Year | Observation Period | *ULAM* BB | *QUAL* BB | *JUNI* BB | *RHTY* BB | *PODE* BB | *SAAL* BB | *QUVE* BB | |
|---|---|---|---|---|---|---|---|---|---|
| 2010 | Modern | 96 | 104.9 | 108.3 | 104.8 | 103.7 | 116.6 | | |
| 2011 | Modern | 116 | 129.9 | 127.2 | 131 | 127.7 | 136 | 128.2 | |
| 2012 | Modern | 92 | 102.9 | 101.7 | 95.1 | 109.4 | 111.4 | 96 | |
| 2013 | Modern | 110.8 | 126 | 124.3 | 126 | 125.5 | 128.5 | 124.9 | |
| 2014 | Modern | 108.4 | 131.6 | 120 | 126.8 | 129 | 134.3 | 131.9 | Average variation in BB DOY |
| | Variation in BB DOY between years | 24 | 28.7 | 25.5 | 35.9 | 25.3 | 24.6 | 35.9 | 28.6 |
