# Supplementary material for: A century of climate warming results in growing season extension: Delayed autumn leaf phenology in north central North America
Source: PLoS One. 2023 Mar 3;18(3):e0282635. doi: 10.1371/journal.pone.0282635 (PMC9983848; doi:10.1371/journal.pone.0282635)
Supplement: S2 File — Yearly foliage coloration DOY observations for the seven focal species in the modern and historic observation periods, Species-specific average foliage coloration DOY for the modern and historic observation periods, and Yearly variation in foliage coloration DOY in the modern observation period. (PDF) [file pone.0282635.s002.pdf]

S2 File. Comparisons of modern versus historic foliage coloration. This file contains yearly and average values for historical and modern observations and interannual variation in foliage coloration DOY among species in the modern period.

S2 Table 1. Yearly foliage coloration DOY observations for the seven focal species in the modern and historic observation periods. Species abbreviations are *ULAM* = *U. americana*, *JUNI* = *J. nigra*, *QUAL* = *Q. alba*, *QUVE* = *Q. velutina*, *PODE* = *P. deltoides*, *RHTY* = *R. typhina*, and *SAAL* = *S. albidum.*

Year	Observation Period	*ULAM* FC	*QUAL* FC	*JUNI* FC	*RHTY* FC	*PODE* FC	*SAAL* FC	*QUVE* FC
2010	Modern	295	301.2	286.8	281	282.4	290.2	302.7
2011	Modern	301.6	300.9	285.7	282.3	287	281.8	306.2
2012	Modern	287.8	297.1	281.8	279.9	289	281	297.2
2013	Modern	303.4	307	287.5	282.4	290.9	288.6	310.2
1883	Historic	277	291	274		278	273	288
1884	Historic	267	287	262	263	260	274	287
1885	Historic	270	277	282	271	273	282	275
1886	Historic	285	296	278	279	283	285	297
1887	Historic	268	283	263	268	283	268	283
1888	Historic	284	292	274	266	272	275	294
1889	Historic	273	297	265	266	281	273	297
1890	Historic	280	297	275	271	297	275	297
1891	Historic	280	281	280	274	286	280	280
1892	Historic	280	282	276	276	289	279	292
1893	Historic	290	290	292	273	290	279	290
1894	Historic	278	288	269	270	288	273	288
1895	Historic	284	301	284	275	295	275	295
1896	Historic	280	280	268	280	275	270	280
1897	Historic	275		273	265	291	275	279
1904	Historic	298						
1905	Historic	291	283	265	273	273	268	274
1906	Historic	289						
1907	Historic	292	298	270		292		292
1908	Historic	279				296	294	
1910	Historic	298						
1912	Historic	285	309			294	285	294

S2 Table 2. Species-specific average foliage coloration DOY for the modern and historic observation periods. The change in average DOY foliage coloration is calculated as the Modern average FC DOY - the Historic average FC DOY for each species.

Observation Period	Average *ULAM* FC	Average *QUAL* FC	Average *JUNI* FC	Average *RHTY* FC	Average *PODE* FC	Average *SAAL* FC	Average *QUVE* FC
Modern	297.0	301.6	285.5	281.4	287.3	285.4	304.1
Historic	282.0	290.1	273.5	271.3	284.0	276.8	287.9
Change in average DOY FC	15.0	11.4	11.9	10.1	3.3	8.6	16.2

S2 Table 3. Yearly variation in foliage coloration DOY in the modern observation period. Variation in FC DOY between years is calculated as the difference between the latest and earliest DOY foliage coloration for each species.

Year	Observation Period	*ULAM* FC	*QUAL* FC	*JUNI* FC	*RHTY* FC	*PODE* FC	*SAAL* FC	*QUVE* FC	
2010	Modern	295	301.2	286.8	281	282.4	290.2	302.7	
2011	Modern	301.6	300.9	285.7	282.3	287	281.8	306.2	
2012	Modern	287.8	297.1	281.8	279.9	289	281	297.2	
2013	Modern	303.4	307	287.5	282.4	290.9	288.6	310.2	Average variation in FC DOY
	Variation in FC DOY between years	15.6	9.9	5.7	2.5	8.5	9.2	13	9.2
