# Supplementary material for: A century of climate warming results in growing season extension: Delayed autumn leaf phenology in north central North America
Source: PLoS One. 2023 Mar 3;18(3):e0282635. doi: 10.1371/journal.pone.0282635 (PMC9983848; doi:10.1371/journal.pone.0282635)
Supplement: S3 File — Yearly leaf fall DOY observations for the seven focal species in the modern and historic observation periods, Species-specific average leaf fall DOY for the modern and historic observation periods, and Yearly variation in leaf fall DOY in the modern observation period. (PDF) [file pone.0282635.s003.pdf]

S3 File. Comparisons of modern versus historic leaf fall. This file contains yearly and average values for historical and modern observations and interannual variation in leaf fall DOY among species in the modern period.

S3 Table 1. Yearly leaf fall DOY observations for the seven focal species in the modern and historic observation periods. Species abbreviations are *ULAM = U. americana*, *JUNI = J. nigra*, *QUAL = Q. alba*, *QUVE = Q. velutina*, *PODE = P. deltoides*, *RHTY = R. typhina*, and *SAAL = S. albidum.*

Year	Observation Period	*ULAM* LF	*QUAL* LF	*JUNI* LF	*RHTY* LF	*PODE* LF	*SAAL* LF	*QUVE* LF
2010	Modern	305.5	315	293.8	291.5	297.1	301.8	315
2011	Modern	312.5	316	290.2	291.3	298.6	299.4	314
2012	Modern	300	306	295	298.1	297.4	298.2	303.3
2013	Modern	314.5	321	298.8	290.4	302.6	302.6	320.3
1883	Historic	298	314	295		298	303	309
1884	Historic	315	325	291	294	300	313	326
1885	Historic	295	317	296	300	295	302	310
1886	Historic	307	312	287	295	297	301	311
1887	Historic	309	305	274	283	293	285	301
1888	Historic	296	313	286	284	287	292	306
1889	Historic	291	303	278	273	285	282	303
1890	Historic	301	314	283	280	303	286	310
1891	Historic	297	318	288	283	304	293	309
1892	Historic	293	296	290	292	296	292	313
1893	Historic	303	321	290	287	309	295	316
1894	Historic	290	301	289	283	309	278	303
1895	Historic	298	310	291	284	303	285	309
1896	Historic	292	298	276	289	287	275	292
1897	Historic	298		279	274	314	285	291
1904	Historic	313						
1905	Historic	301	308	295	295	301	283	295
1907	Historic	303	309	278		303		303
1908	Historic	299						
1910	Historic	314						
1912	Historic	316	314	288		311	294	302

S3 Table 2. Species-specific average leaf fall DOY for the modern and historic observation periods. The change in average DOY leaf fall is calculated as the Modern average LF DOY - the Historic average LF DOY for each species.

Observation Period	Average *ULAM* LF	Average *QUAL* LF	Average *JUNI* LF	Average *RHTY* LF	Average *PODE* LF	Average *SAAL* LF	Average *QUVE* LF
Modern	308.1	314.5	294.5	292.8	298.9	300.5	313.2
Historic	301.4	310.5	286.3	286.4	299.7	290.8	306.1
Change in average DOY LF	6.7	4.0	8.1	6.4	-0.8	9.7	7.1

S3 Table 3. Yearly variation in leaf fall DOY in the modern observation period. Variation in LF DOY between years is calculated as the difference between the latest and earliest DOY leaf fall for each species.

Year	Observation Period	*ULAM* LF	*QUAL* LF	*JUNI* LF	*RHTY* LF	*PODE* LF	*SAAL* LF	*QUVE* LF	
2010	Modern	305.5	315	293.8	291.5	297.1	301.8	315	
2011	Modern	312.5	316	290.2	291.3	298.6	299.4	314	
2012	Modern	300	306	295	298.1	297.4	298.2	303.3	
2013	Modern	314.5	321	298.8	290.4	302.6	302.6	320.3	Average variation in LF DOY
	Variation in FC DOY between years	14.5	15	8.6	7.7	5.5	4.4	17	10.4
