# Supplementary material for: A century of climate warming results in growing season extension: Delayed autumn leaf phenology in north central North America
Source: PLoS One. 2023 Mar 3;18(3):e0282635. doi: 10.1371/journal.pone.0282635 (PMC9983848; doi:10.1371/journal.pone.0282635)
Supplement: S4 File — Yearly growing season lengths (GSL, days) for the seven focal species in the modern and historic observation periods, Species-specific average growing season length (days) for the modern and historic observation periods, and Yearly variation in total GSL in the modern observation period. (PDF) [file pone.0282635.s004.pdf]

S4 File. Comparisons of modern versus historic growing season length (GSL). This file contains yearly and average values for historical and modern observations and interannual variation in total GSL (days) among species in the modern period.

S4 Table 1. Yearly growing season lengths (GSL, days) for the seven focal species in the modern and historic observation periods. GSL is defined as the difference in days between the foliage coloration DOY and the budburst DOY. Species abbreviations are *ULAM* = *U. americana*, *JUNI* = *J. nigra*, *QUAL* = *Q. alba*, *QUVE* = *Q. velutina*, *PODE* = *P. deltoides*, *RHTY* = *R. typhina*, and *SAAL* = *S. albidum.*

Year	Observation Period	*ULAM* GSL	*QUAL* GSL	*JUNI* GSL	*RHTY* GSL	*PODE* GSL	*SAAL* GSL	*QUVE* GSL
2010	Modern	199.0	196.3	178.6	176.2	178.7	173.6	
2011	Modern	185.6	171.0	158.5	151.3	159.3	145.8	178.0
2012	Modern	195.8	194.2	180.1	184.8	179.6	169.6	201.2
2013	Modern	192.6	181.0	163.2	156.4	165.4	160.1	185.3
1883	Historic	170	163	142		163	143	155
1884	Historic	159	160	135	137	140	147	159
1885	Historic	157	144	143	130	159	146	139
1886	Historic	179	182	160	166	171	174	183
1887	Historic	162	153	123	140	154	136	158
1888	Historic	168	160	141	125	148	141	162
1889	Historic	162	170	145	145	153	154	169
1890	Historic	174	171	150	139	165	158	173
1891	Historic	169	161	160	161	171	162	161
1892	Historic	169	145	141	143	167	158	157
1893	Historic	184	157	158	141	167	153	158
1894	Historic	161	162	146	138	171	153	165
1895	Historic	168	175	154	153	174	151	172
1896	Historic	170	163	151	169	165	159	164
1897	Historic	161		147	141	171	151	152
1912	Historic	171	181			177		167

S4 Table 2. Species-specific average growing season length (days) for the modern and historic observation periods. The change in average GSL is calculated as the Modern average GSL - the Historic average GSL for each species.

Observation Period	Average *ULAM* GSL	Average *QUAL* GSL	Average *JUNI* GSL	Average *RHTY* GSL	Average *PODE* GSL	Average *SAAL* GSL	Average *QUVE* GSL	
Modern	193.3	185.6	170.1	167.2	170.7	162.3	188.2	
Historic	167.8	163.1	146.4	144.9	163.5	152.4	162.1	Average
Change in average GSL (days)	25.5	22.5	23.7	22.3	7.2	9.9	26.0	19.6

S4 Table 3. Yearly variation in total GSL in the modern observation period. Variation in GSL between years is calculated as the difference between the longest and shortest GSL for each species.

Year	Observation Period	*ULAM* GSL	*QUAL* GSL	*JUNI* GSL	*RHTY* GSL	*PODE* GSL	*SAAL* GSL	*QUVE* GSL	
2010	Modern	199.0	196.3	178.6	176.2	178.7	173.6		
2011	Modern	185.6	171.0	158.5	151.3	159.3	145.8	178.0	
2012	Modern	195.8	194.2	180.1	184.8	179.6	169.6	201.2	
2013	Modern	192.6	181.0	163.2	156.4	165.4	160.1	185.3	Average variation in GSL
	Variation in GSL between years	13.4	25.3	20.0	33.5	20.4	27.8	23.2	23.4
